# Dissolved-oxygen feedback control fermentation for enhancing β-carotene in engineered *Yarrowia lipolytica*

**DOI:** 10.1038/s41598-020-74074-0

**Published:** 2020-10-13

**Authors:** Peng Jun Lv, Shan Qiang, Liang Liu, Ching Yuan Hu, Yong Hong Meng

**Affiliations:** 1 Engineering Research Center of High Value Utilization of Western China Fruit Resources, Ministry of Education, National Research and Development Center of Apple Processing Technology, College of Food Engineering and Nutritional Science, Shaanxi Normal University, 620 West Changan Avenue, Changan, Xian 710119 P.R. China; 2 Xian Healthful Biotechnology Co., Ltd., Hang Tuo Road, Changan, Xi’an, 710100 People’s Republic of China

**Keywords:** Biological techniques, Biotechnology

## Abstract

The DO-stat fed-batch fermentation was carried out to explore the volumetric productivity of β-carotene in engineered *Yarrowia lipolytica* C11 strain. Using DO-stat fed-batch fermentation, we achieved 94 g/L biomass and 2.01 g/L β-carotene. Both biomass and β-carotene were about 1.28-fold higher than that in fed-batch fermentation. The ATP, NADP^+^/NADPH, and gene expression levels of *tHMG*, *GGS1*, *carRA*, and *carB* were promoted as compared to that in fed-batch fermentation. As for as the kinetic parameters in DO-stat fed-batch fermentation, μ_m_′, Y_x/s_′, and Y_p/s_′ was 0.527, 0.353, and 0.158, respectively. The μ_m_′ was elevated 4.66-fold than that in fed-batch fermentation. These data illustrate that more dissolved oxygen increased the biomass. The Y_x/s_′ and Y_p/s_′ were increased 1.15 and 22.57-fold, which suggest that the DO-stat fed-batch fermentation reduced the Crabtree effect and improved the utilization rate of glucose. Therefore, DO-stat fed-batch fermentation is a promising strategy in the industrialized production of β-carotene.

## Introduction

β-Carotene (C_40_H_56_), one of many carotenoids, is the precursor of vitamin A^1,2^. β-carotene has become an essential ingredient in food additives, cosmetics, and pharmaceuticals^3,4^. β-carotene has the function of enhancing immune functions, anti-oxidation, and anti-cancer activities^5–8^. The global β-carotene market size is anticipated to reach USD 583 million by the end of 2024^9^. Currently, the primary sources of β-carotene include chemical synthesis, plant extracts, and microbial fermentation. Among these, the microbial fermentation is considered to be the best way to meet market demands, as it is environment-friendly and economical.

The most commonly used microorganisms to produce β-carotene are *Blakeslea trispora*^10^, engineered *Yarrowia lipolytica*^11^, *Saccharomyces cerevisiae*^12^, and *Escherichia coli*^13^. β-carotene is a lipid-soluble compound and stored in the lipid bodies of engineered oleaginous yeast *Y. lipolytica*. Compared with the non-oleaginous *Saccharomyces* cerevisiae or *Escherichia coli*, *Y. lipolytica* is more suitable for the production of β-carotene. *Y. lipolytica* is a “generally recognized as safe” strain and a potential industrial host for the production of bio-ingredients^14,15^. An engineered *Y. lipolytica* strain to maximize β-carotene production and 1.47 mg/L of β-carotene was obtained after six days of cultivation in a flask^16^. Another engineered *Y. lipolytica* strain was constructed by incorporating two genes, bi-functional phytoene synthase/lycopene cyclase (*crtYB*) and phytoene desaturase (*crtI*) from the red yeast *Xanthophyllomyces dendrorhous*. After 72 h cultivation, 31.1 mg/L β-carotene was obtained^17^. Although β-carotene-producing engineered *Y. lipolytica* has made significant progress, insufficient oxygen supply during fermentation has not been systematically optimized.

The production of β-carotene in *Y. lipolytica* requires aerobic culture. When the glucose concentration in the culture medium is high, the Crabtree effect often occurs in aerobic conditions during most yeast fermentation, leading to the production of alcohol and acetate through substrate-level phosphorylation^18,19^. Excessive alcohols and acids compete with β-carotene synthesis for the substrate of acetyl-CoA. The Crabtree effect eventually leads to a reduction in the yield of the target product^20^. Fed-batch cultures provide a carbon source at a low level by feeding essential nutrients incrementally^21^. Therefore, this culturing technique is used to overcome the Crabtree effect^22^. Fed-batch strategies include constant dissolved oxygen value feeding (DO-stat)^23–27^, constant specific growth rate feeding (μ-stat)^25,28^, constant pH feeding (pH–stat)^20,29,30^, and constant carbon source concentration feeding^31^. Besides the Crabtree effect, oxygen in short supply is another obstacle in aerobic culture.

With cells growing, oxygen consumption exceeds the maximum oxygen transfer capacity, which becomes a limiting factor for cell growth^32^. Thus, a solution for microbes to receive an adequate amount of oxygen is by decreasing the specific growth rate and amount of oxygen consumption. Usually, DO-stat strategy can control dissolved oxygen at a constant value using fed substrate at a specific rate. Once the carbon source is exhausted in the logarithmic growth phase, the O_2_ value rapidly increases due to cell death from hypoxia. If the carbon source was fed timely, the O_2_ value decreases as the cells re-utilize carbon source and restore growth. Subsequently, a constant DO level can be maintained by continuous feeding and keep a balance between oxygen consumption and supply. The DO-stat strategy typically works well in a defined media where nutrient depletion results in cell death and a rapid elevating DO^33^. Many authors have used the DO-stat feeding strategy to achieve high yield. The DO-stat fed-batch fermentation strategy was used to produce tyrosine phenol lyase by recombinant *Escherichia coli*, the final biomass was 35.6 g/L, and the volumetric activity reached 12,292 U/L after 30 h cultivation^34^. The DO-stat feeding strategy was promising together with the use of ammonium hydroxide for pH control to improve P(3HB) volumetric productivity^35^. This particular method has been widely used in the aerobic culture to produce highly valuable chemicals and biofuels.

The engineered *Y. lipolytica* stain YL-C11 (matA, leucine^+^, uracil^+^, xpr2-322, axpl^-^, Δku70, Δ*snf*:: *tHMG*-*carB*-*carRA*-*ggs1*, Δgut2:: *did2*-*ura3*) was used to produce β-carotene. The purpose of this study was to develop a DO-stat culture strategy to improve biomass and β-carotene yield of YL-C11. The changes in ATP, NADP^+^/NADPH, and gene expression level during the culture process were also explored. The specific kinetic models relating the cell growth to the limiting substrate (glucose) and primary product (β-carotene) were constructed. This study established an effective method to increase the yield of β-carotene and provides a new vehicle for industrialized β-carotene production.

## Results

### Fed-batch fermentation

**Figure 1:**
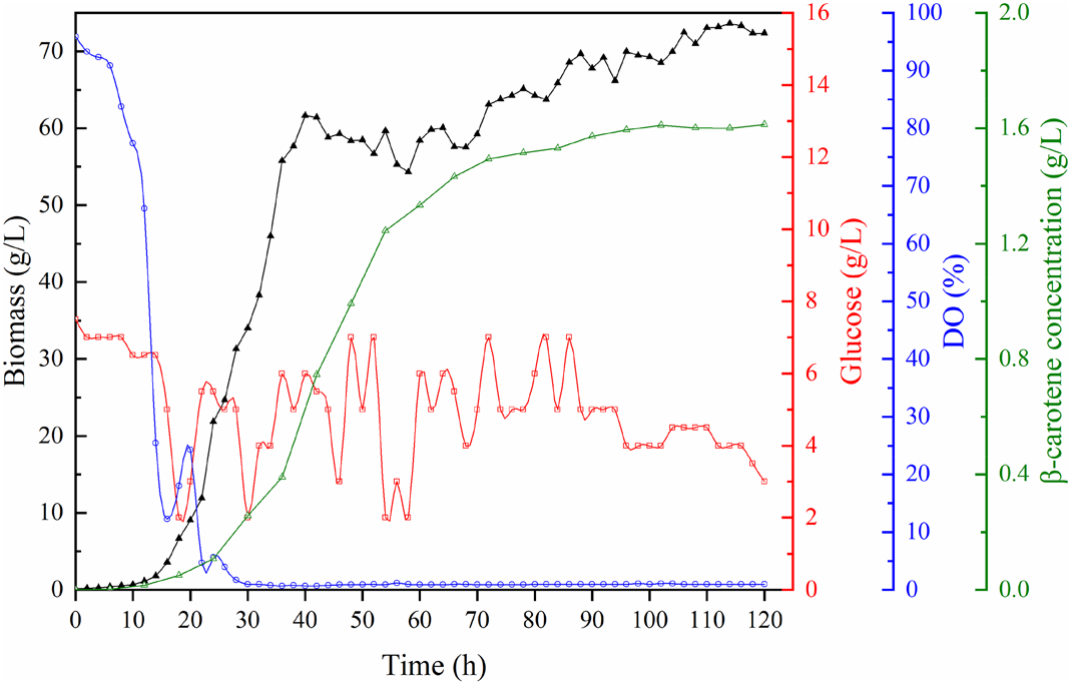
The time-course of dry cell weight, dissolved oxygen, glucose concentration, and β-carotene concentration during fed-batch fermentation for YL-C11. The glucose concentration was fed at around 5 g/L.

Fed-batch experiments were performed with glucose maintained at around 5 g/L to determine the growth characteristics of YL-C11. Cell growth experienced a short lag period after the inoculation (Fig. 1). Cell growth entered the logarithmic growth phase, and biomass increased rapidly during the 12–42 h fermentation. After 42 h fermentation, biomass increased slowly, and cell growth entered the stationary period. Although cell growth was in a stationary phase, the synthesis of β-carotene continued. It was not until the 102 h of fermentation that the synthesis of the β-carotene trend finally stopped. Glucose (1200 g) was fed to maintain glucose concentration at around 5 g/L. On-line monitoring results show that the DO value decreased rapidly in the first 28 h of fermentation and reached 0% when the fermentation time extended from 28 to 120 h. Cell biomass reached 73.5 g/L, and β-carotene concentration reached 1.6 g/L at the end of fermentation.

### DO-stat fed-batch fermentation

**Figure 2:**
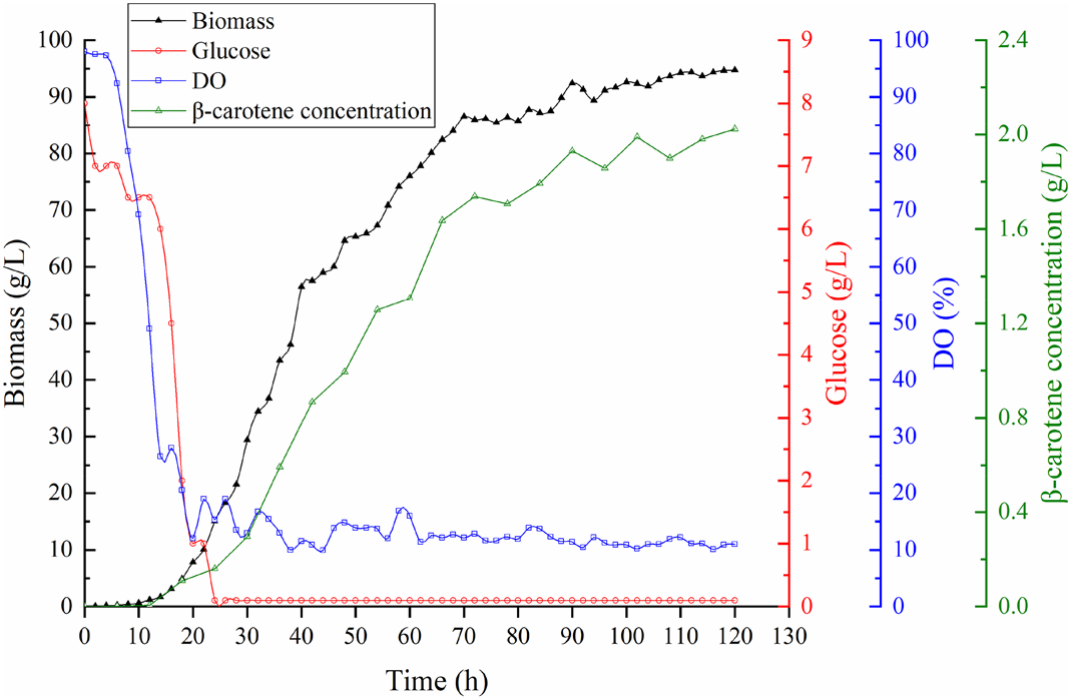
The time-course of dry cell weight, dissolved oxygen, glucose concentration, and β-carotene concentration during DO-stat fed-batch fermentation for YL-C11. The DO-stat fed-batch induction was initiated at 22 h, and the DO was controlled around 10–20%.

We conducted DO-stat fed-batch fermentation to increase the yield of β-carotene (Fig. 2). This strategy is based on online feedback of DO value, which tends to increase upon when the substrate was depleted completely, thus signaling the automatic feeding of glucose to the fermenter. Glucose (1350 g) was used to maintain DO value at 10–20%. The medium for the fed-batch phase contained 8 g/L glucose, which was depleted completely after 20 h. DO-stat fed-batch fermentation was implemented at this time until the end of fermentation. Figure 2 shows that cell growth had entered the logarithmic phase after a 12 h lag period. The increase of biomass tended to be stable after 96 h fermentation, which demonstrates the cell growth entered the stationary period. 94 g/L biomass and 2.01 g/L β-carotene concentration were harvested at the end of DO-stat fed-batch fermentation, separately. 8 g ammonium sulfate was fed into 5 L fermentation broth at 24, 48, and 72 h to avoid pH rise, separately.

Application of DO-stat fed-batch fermentation resulted in 1.28-fold higher production of biomass and the β-carotene production, respectively. More glucose and ammonium sulfate were consumed in DO-stat fed-batch fermentation.

### More ATP and NADP^+^/NADPH were produced in DO-stat fed-batch fermentation

The intracellular levels of ATP content and NADP^+^/NADPH were determined to explore the effect of feeding strategy on the generation of energy ATPs and NADP^+^/NADPH.

**Figure 3:**
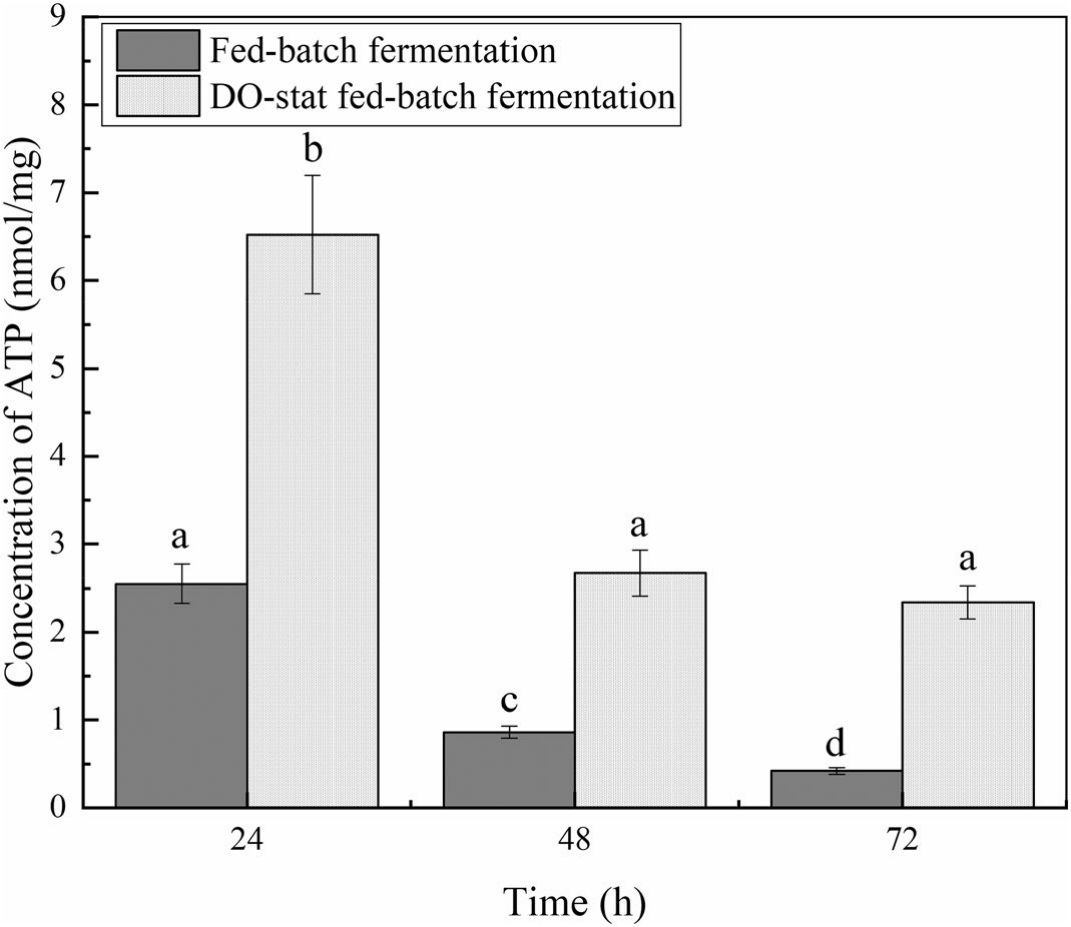
More ATP was determined in DO-stat fed-batch fermentation. The ATP of both fermentations was extracted and assayed, as described in the text. Error bars represent ± SD. The date with a different superscript letter is significantly different at *P* < 0.05 (ANOVA).

ATP was gradually diminished during fed-batch fermentation. The highest level of ATP appeared at 24 h during the fed-batch fermentation (Fig. 3). At the 48 h, the ATP content was dropped to 0.86 nM/mg protein. The ATP content was only 0.42 nM/mg protein at the 72 h.

More ATP were generated in the DO-stat strategy when compared to the fed-batch strategy. The ATP content was 6.52 nM/mg protein in the initial stage of the logarithmic phase at DO-stat fed-batch fermentation. ATP content gradually decreased as the fermentation progressed and dropped to 2.34 nM/mg protein at the 72 h.

**Figure 4:**
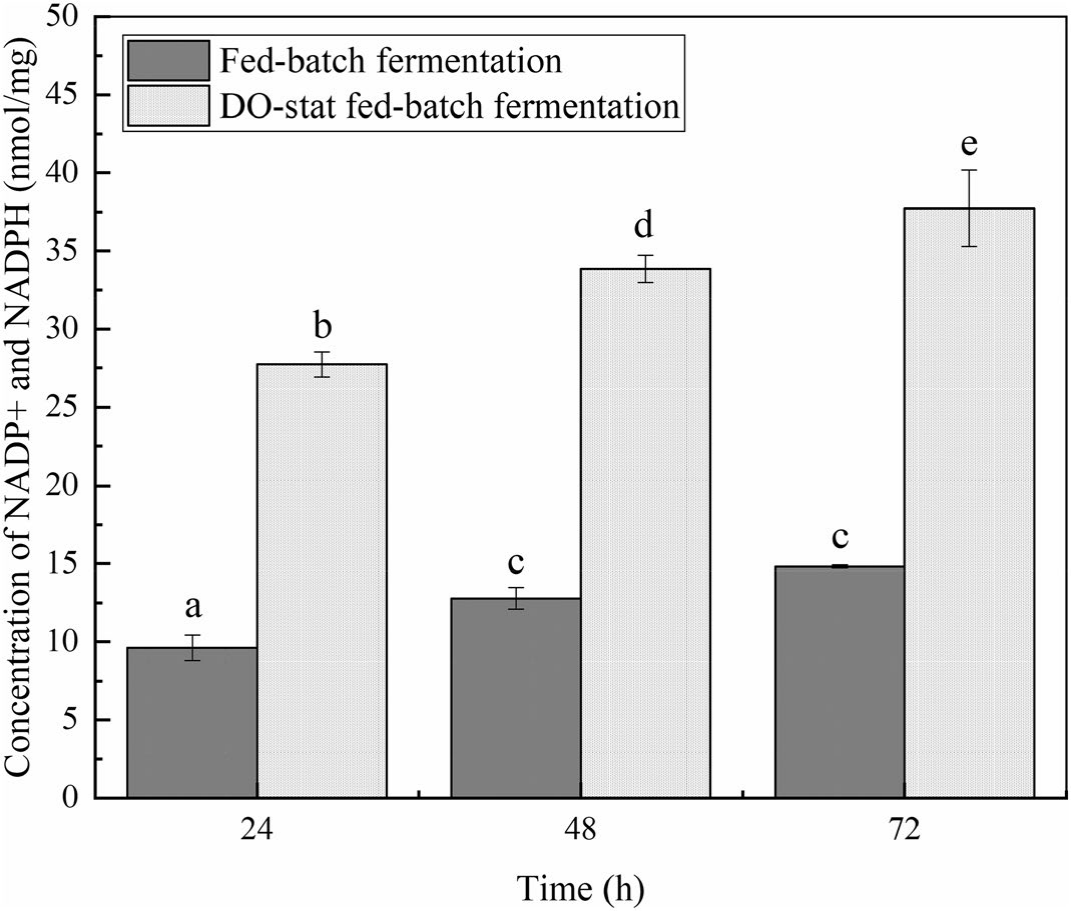
More NADP^+^/NADPH was determined in DO-stat fed-batch fermentation. The NADP^+^/NADPH of both fermentations was extracted and assayed, as described in the text. Error bars represent ± SD. The date with a different superscript letter is significantly different at *P* < 0.05 (ANOVA).

More NADP^+^/NADPH was generated during the fed-batch fermentation (Fig. 4). NADP^+^/NADPH content was the least at the initial stage of the logarithmic phase in fed-batch fermentation. At 48 h, the NADP^+^/NADPH content reached 12.79 nM/mg protein. The NADP^+^/NADPH content was 14.82 nM/mg protein at 72nd of fed-batch fermentation.

More NADP^+^/NADPH were generated in DO-stat fermentation when compared to fed-batch fermentation. NADP^+^/NADPH content was least in the initial stage of the logarithmic phase during DO-stat fed-batch fermentation. NADP^+^/NADPH content was increased to 33.84 nM/mg protein at the 48 h. NADP^+^/NADPH content reached 37.72 nM/mg protein at the 72 h during DO-stat fed-batch fermentation.

The ATP and NADP^+^/NADPH analysis results show that the ATP and NADP^+^/NADPH content under high oxygen conditions was higher than under low oxygen conditions. The content of ATP exhibited a downward trend, and the content of NADP^+^/NADPH show an upward trend during the entire fermentation in both fed types.

### Transcriptional level of related genes in the β-carotene biosynthesis pathway was higher in DO-stat fed-batch fermentation

*tHMG*, *GGS1*, *carRA,* and *carB* are four crucial genes in the β-carotenoid synthesis pathway (see Supplementary Fig S2). The transcription levels of *tHMG*, *GGS1*, *carRA*, and *carB* were separately studied in the YL-C11 strain to clarify the regulatory mechanism of both fermentation strategies.

**Figure 5:**
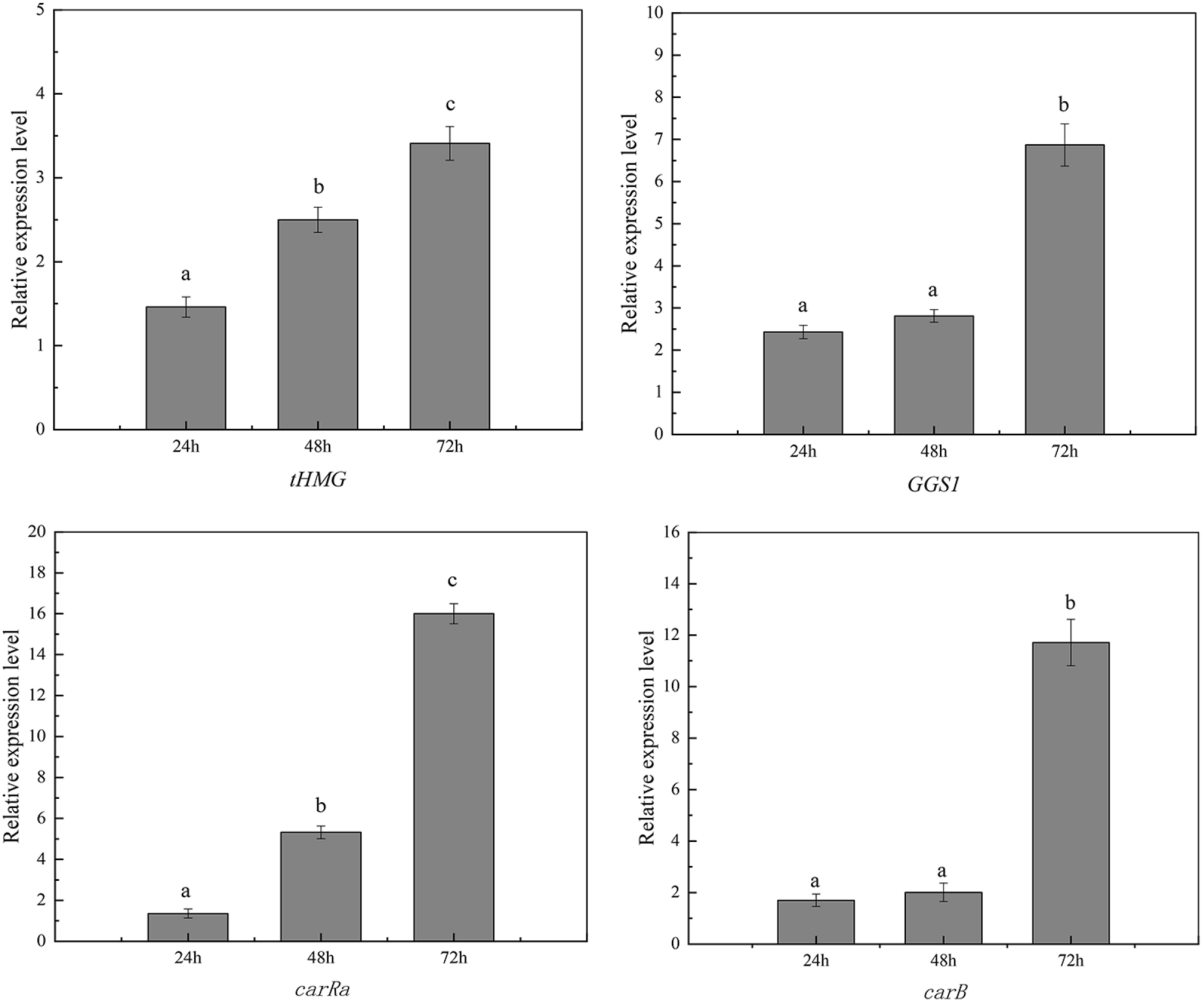
Higher transcriptional level of related genes in the β-carotene biosynthesis pathway at DO-stat fed-batch fermentation. Error bars represent ± SD. The date with a different superscript letter is significantly different at *P* < 0.05 (ANOVA).

In carotenogenesis pathways, gene expression levels of *tHMG*, *GGS1*, *carRA*, and *carB* were higher under high DO conditions than under low DO conditions. We used the transcription levels of fed-batch fermentation as 1 in Fig. 5. At the 24, 48, and 72 h, the transcription levels of *tHMG* in DO-stat strategy were increased by 1.5, 2.4, and 3.4-fold, respectively, when compared to fed-batch fermentation. Similarly, the transcription levels of *GGS1* in DO-stat fermentation strategy were increased by 2.4, 2.8, and 6.9 times, respectively, compared to fed-batch fermentation at 24, 48, and 72 h. The transcription levels of *carRA* in the DO-stat strategy were 1.4, 5.3, and 16 times higher than that of fed-batch fermentation at 24, 48, and 72 h, respectively. The transcription levels of *carB* in the DO-stat strategy were 1.7, 2.1, and 11.7 times higher than that of fed-batch fermentation at 24, 48, and 72 h, respectively.

### Fermentation kinetics of YL-C11

The specific kinetic models were used to describe the fermentation process, including cell growth, product formations, and substrate consumption. Establishing a system model to investigate cell growth in all kinds of fermentation environments would be well utilized in directing scale production. Although the feed will slightly increase the volume of the fermentation broth, the volume of the fermentation broth was considered to be constant due to sampling in this study.

### Kinetics of cell growth

**Figure 6:**
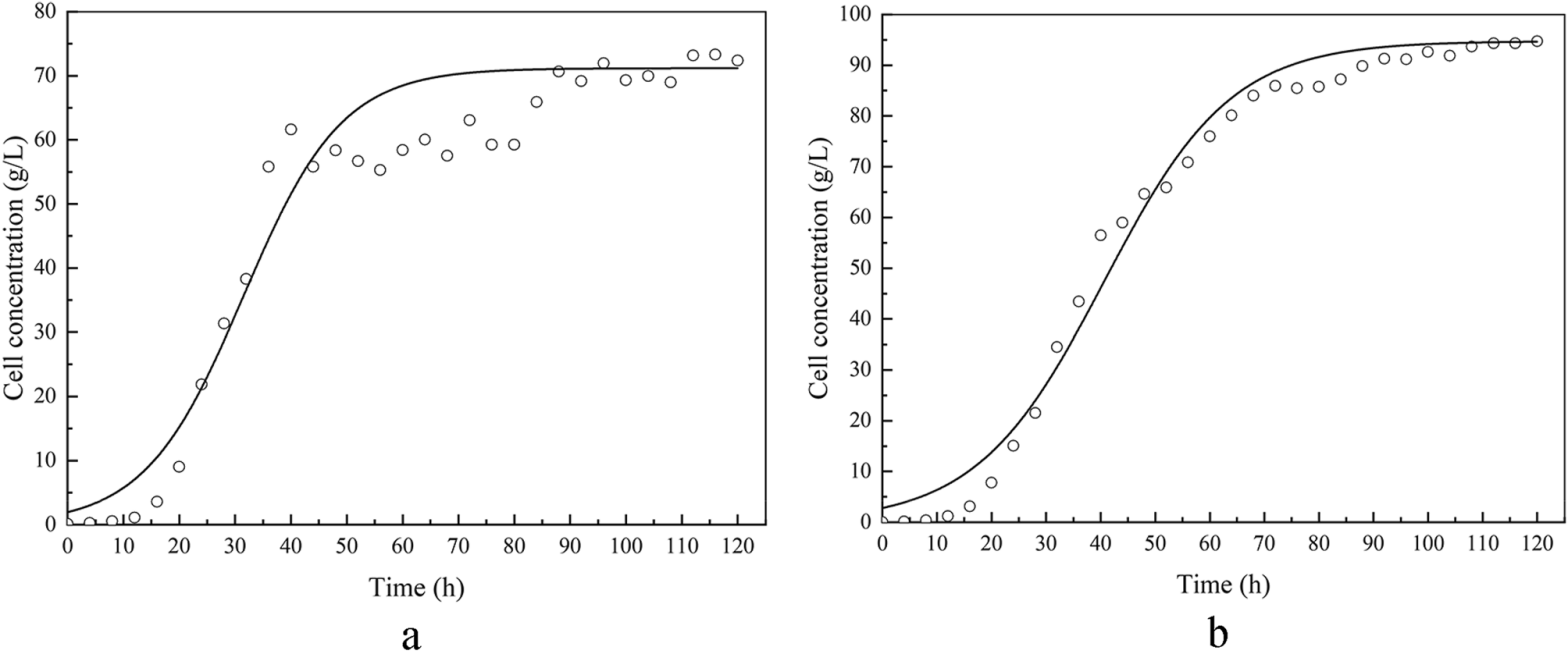
Simulations of kinetic models and experimental results of biomass at fed-batch and DO-stat fed-batch fermentation (**a** fed-batch fermentation; **b** DO-stat fed-batch fermentation; Solid line represents fitted data and circles represent experimental data).

Figure 6 shows the time courses of biomass separately derived from the model equations and the experiments. Figure 6a,b show the fitting results from the fed-batch fermentation and DO-stat fed-batch fermentation, respectively. The cell growth shows an “S” pattern. The cell growth curve of YL-C11 was consistent with the logistic equation, which was presented as follows:

1 $$\frac{dX}{dt}={\mu }_{m}\left(1-\frac{X}{{X}_{m}}\right)X$$

where X is the biomass concentration (g/L), t is fermentation time (h), dX/dt is cell growth rate [g/(L h)]. μ_m_ (h^−1^) is the maximum specific growth rate in fed-batch fermentation and μ_m_′ (h^−1^) is the maximum specific growth rate in DO-stat fed-batch fermentation. X_m_ (g/L) is the maximum microorganism concentration in fed-batch fermentation and X_m_′ (g/L) is the maximum microorganism concentration in DO-stat fed-batch fermentation.

The simulated kinetic parameters were listed in Table 1. The models fit the experimental data well with R^2^ values of 0.9315 for fed-batch fermentation and 0.9526 for DO-stat fed-batch fermentation, indicating the adequacy of the logistic equation model to fit experimental data. The μ_m_′ value in DO-stat fed-batch fermentation is 0.527, which is 4.66 times higher than μ_m_ in fed-batch fermentation (0.113). This result suggests that DO-stat fed-batch fermentation resulted in a higher cell growth rate; thus, more biomass was obtained.

**Table 1:** Kinetic parameters of cell growth, β-carotene production, and glucose consumption.

	Parameters of cell growth			Parameters of β-carotene production			Parameters of glucose consumption			
Fed-batch fermentation	X_m_	μ_m_	R^2^	α	β	R^2^	Y_x/s_	Y_p/s_	m	R^2^
	71.18	0.113	0.9315	0.0216	1.52 × 10^–5^	0.9981	0.306	0.007	–	–
DO-stat fed-batch fermentation	X_m_′	μ_m_′	R^2^	α′	β′	R^2^	Y_x/s_′	Y_p/s_′	m′	R^2^
	94.73	0.527	0.9526	− 0.5876	0.2673	0.9505	0.353	0.158	− 1.6912	0.9758

### Kinetics of β-carotene synthesis

There are three fermentation forms: I, growth-related type; II, growth part-related type; III, non-growth-related type. In general, the formation of weak organic acids, such as citric acid, lactic acid, and succinic acid, by microbial fermentation, has been well simulated by the Luedeking–Piret model that consists of a growth-associated part and non-growth-associated part. In this case, we employed this model to describe the kinetics of β-carotene production.

2 $$\frac{dP}{dt}=\alpha \frac{dX}{dt}+\beta X$$

where P is the product concentration, X (g/L) is the concentration of cells, t is the fermentation time, α and β are the coefficients. α (g/g) denotes the parameters for product formation constant (associated with the cell growth rate) in fed-batch fermentation and α′ (g/g) denotes the parameters for product formation constant (associated with the cell growth rate) in DO-stat fed-batch fermentation. β (g/g) denotes the parameters for product formation constant (related to the number of cells) in fed-batch fermentation and β′ (g/g) denotes the parameters for product formation constant (related to the number of cells) in DO-stat fed-batch fermentation. As for three fermentation types: I: α ≠ 0, β = 0; II: α ≠ 0, β ≠ 0; III: α = 0, β ≠ 0.

**Figure 7:**
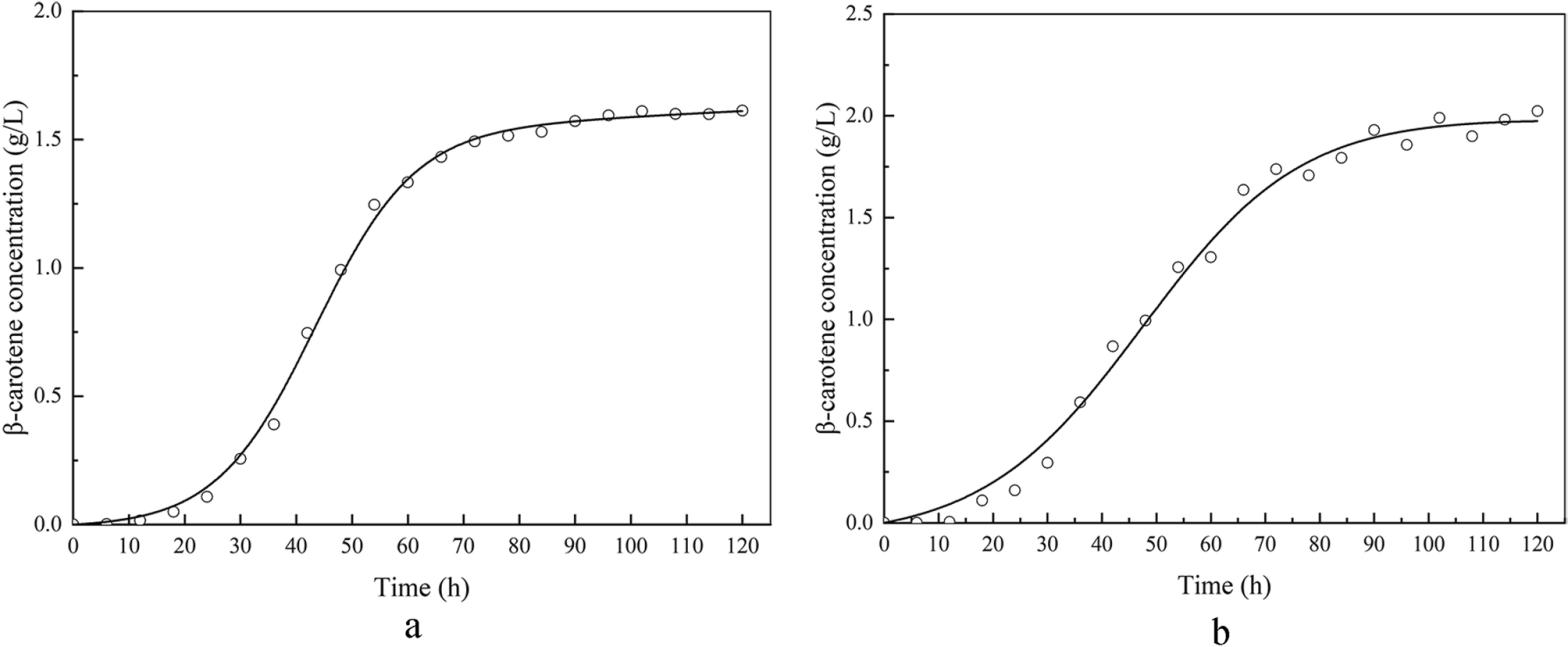
Simulations of kinetic models and experimental results β-carotene synthesis at fed-batch and DO-stat fed-batch fermentation (**a** fed-batch fermentation; **b** DO-stat fed-batch fermentation; Solid line represents fitted data and circles represent experimental data).

Figure 7 depicts the time courses of β-carotene content separately derived from the model equations and the experiments. Figure 7a,b show the fitting results for the fed-batch fermentation and DO-stat fed-batch fermentation, respectively. β-carotene synthesis of engineered *Y. lypolitica* belongs to a partial coupling with cell growth (fermentation type II) because the rate of product formation is related to both the growth rate and the number of cells. A mathematical model was proposed by Luedeking and Piret, which properly described the mechanism of β-carotene synthesis.

The simulated kinetic parameters were listed in Table 1. It shows that a relatively accurate result could be observed between experimental and simulation data. The value of α was 0.0216 for fed-batch fermentation, and α′ was − 0.5876 for DO-stat fed-batch fermentation, separately. The value of β was 1.52 × 10^–5^ for fed-batch fermentation, and β′ was 0.2673 for DO-stat fed-batch fermentation, separately.

A higher α value was obtained for the fed-batch fermentation, indicating the β-carotene synthesis was mainly affected by cell growth rate at fed-batch fermentation. A higher β′ value was obtained for DO-stat fed-batch fermentation, which indicates the β-carotene synthesis was affected mainly by the number of cells during DO-stat fed-batch fermentation.

**Figure 8:**
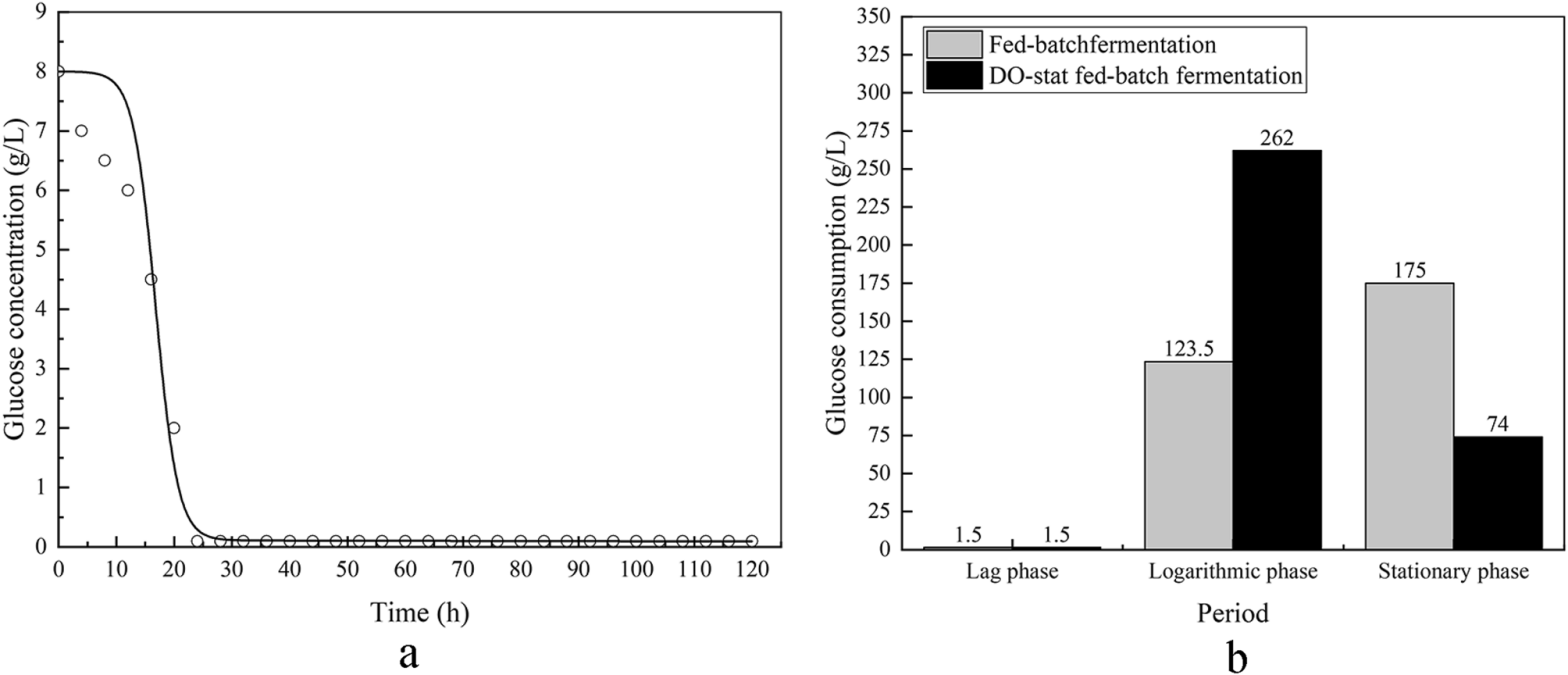
Simulation of kinetic models and experimental results of glucose concentration (**a**) (Solid line represents fitted data and circles represent experimental data) and glucose consumption rate in different periods (**b**).

### Kinetics of glucose consumption

Figure 8a shows the nonlinear fitting of experimental data from DO-stat fed-batch fermentation substrate consumption. The classical kinetic model suggested by Luedeking and Piret was chosen to describe the substrate consumption as follows:

3 $$-\frac{dS}{dt}=\frac{1}{{Y}_{X/S}}\frac{dX}{dt}+\frac{1}{{Y}_{P/S}}\frac{dP}{dt}+mX$$

where Y_x/s_ (g/g) and Y_p/s_ (g/g) represent the substrate yield for biomass and product in fed-batch fermentation, respectively. Y_x/s_′ (g/g) and Y_p/s_′ (g/g) represent the substrate yield for biomass and product in DO-fed-batch fermentation, respectively. m and m′ represents the maintenance coefficient in fed-batch fermentation and DO-fed-batch fermentation, respectively. The whole formula (3) represents glucose used to generate biomass, product, and cellular maintenance energy. The simulated kinetic parameters were listed in Table 1. The Eq. (3) fits the DO-state fed-batch fermentation data with an R^2^ of 0.9758. The Y_x/s_′ and Y_p/s_′ are 0.353 and 0.158, respectively. As for fed-batch fermentation, it is difficult to fit the data of glucose consumption because of fluctuations resulted from continuous feeding glucose. The Y_x/s_ is 0.306 in fed-batch fermentation that directly calculated by Y_x/s_ = ∆X/∆S. The Y_p/s_ is 0.007 in fed-batch fermentation solved by Y_p/s_ = ∆P/∆S. Contrasted with fed-batch fermentation, the Y_x/s_′ was 1.15 times higher than Y_x/s_, and the Y_p/s_′ was 22.57 times higher than Y_p/s_. The results demonstrate that more glucose was utilized in DO-stat fed-batch fermentation, and more substrate yield for biomass and product was obtained.

Figure 8b depicts the glucose consumption in different growth periods at fed-batch fermentation and DO-stat fed-batch fermentation. We divided the glucose consumption into three phases: the lag phase, the logarithmic phase, and the stationary phase. In the lag phase, 1.5 g of glucose were consumed in one liter of fermentation broth at both fermentations. In the logarithmic phase, one liter of fermentation broth consumed 262 g glucose for the DO-stat fed-batch fermentation, a 2.12-fold higher than that in fed-batch fermentation. This result clearly shows that more glucose was consumed during the logarithmic phase in DO-stat fed-batch fermentation. In the stationary phase, 74 g glucose was consumed in one liter of fermentation for the DO-stat fed-batch fermentation. Glucose consumption represents a decrease of about 42% when compared to fed-batch fermentation. The lower glucose consumption at the stationary of DO-stat fed-batch fermentation might attribute to shorter stationary phase and the inhibition of Crabtree effects. Inhibited Crabtree effect resulted in less glucose being used to by-products and more substrate utilized to biomass and products.

## Discussion

Oxygen plays a pivotal role in aerobic fermentation. Aerobic microorganisms generally require large amounts of oxygen to generate NAD(P)H or FADH_2_ and ATP for metabolism. Several studies also showed that the dissolved oxygen levels directly affect the synthesis of different enzymes and results in the changes in cell metabolism, product yield, and productivity^36,37^.

The DO level was maintained at 10–20% during DO-stat fed-batch fermentation in this study, and the DO level was significantly higher than that of fed-batch fermentation. We found that different dissolved oxygen level affects the metabolism of YL-C11. Low DO resulted in less ATP and the reducing power [NAD(P)H] that required for cell maintenance and growth in fed-batch fermentation. Using the DO-stat method revealed that biomass was enhanced than that of in fed-batch fermentation. The glycolytic, hexose monophosphate, and tricarboxylic acid cycle (TCA cycle) pathways are the essential pathways to produce energy during metabolism. In DO-stat fed-batch fermentation, the above three pathways were strengthened with adequate oxygen delivery. The oxidative phosphorylation and substrate-level phosphorylation was enhanced simultaneously. We set the oxygen level at 10–20% for DO-stat fed-batch fermentation. That lead to more ATP and NADP^+^/DADPH were generated, thereby resulting in the boosted of biomass and β-carotene concentration. Several studies have confirmed that high oxygen levels could improve the metabolic flux of aerobic microorganisms; thus, more biomass and target product were obtained^34,36,38^.

The main aim of the present investigation was to achieve high cell concentration, thus increase β-carotene concentration. A series of the kinetic models were constructed to elaborate on the relationship among cell growth, substrates, and products. The kinetic parameters were determined from different feeding strategies in this study. These simulations provided an insight into the operational protocol that may be implemented to obtain the best results.

Both the biomass and productivity were increased by 28% at the DO-stat fed-batch fermentation. In contrast, the fed-batch fermentation produced of 73.5 g/L cell biomass and productivity of 0.61 g/L/h. The fermentative capacity was strongly affected by the specific growth rate of aerobic cultures^32^. The simulation results showed that the higher μ_m_′, Y_x/s_′, and Y_p/s_′ values were achieved for DO-stat fed-batch fermentation. The higher μ_m_′ indicates that the cell has a higher growth rate. The higher Y_x/s_′ and Y_p/s_′ indicate that more glucose was used to produce the target product. In addition, the DO-stat feeding strategy extended the logarithmic period, and more glucose was consumed in this period. As a result, more biomass was obtained for the DO-stat fed-batch fermentation. The improved growth can be attributed to that the Crabtree effect was blocked with DO-stat feeding strategy. The Crabtree effects should be strongly avoided in industrial operations as its inhibitory effect in the fermentation pathway by an end product of aerobic glucose utilization. The Crabtree effects are prevented from occurring during DO-stat fed-batch fermentation, which is one of the critical reasons that longer logarithmic phase, higher biomass, and glucose utilization were obtained. Thus, DO-stat fed-batch fermentation is an excellent approach to produce β-carotene. We could use this kinetic model to understand the relationship between cell growth, substrate consumption, and product synthesis for different feeding strategies.

Oxygen plays an important role in aerobic fermentation. Adequate oxygen allows aerobic microorganisms to have the optimal conditions for their growth. In this study, DO-stat fed-batch fermentation was successfully applied to increase dissolved oxygen in broth and the volumetric productivity of β-carotene in an engineered *Y. lipolytica* strain. This study also helped us clarify that changes of dissolved oxygen levels during fermentation have a profound effect on cell physiology and hence viable biomass and production yield. The kinetic model revealed that the DO-stat fed-batch fermentation could make a significant contribution to the industrialized β-carotene production, which will partly promote meeting the commercial β-carotene demand.

## Materials and methods

### Strains and culture conditions

Engineered *Y. lipolytica *stain YL-C11 (matA, leucine+, uracil+, xpr2-322, axpl-, Δ*ku*70, Δ*snf*:: *tHMG*-*carB*-*carRA*-*ggs1*, Δ:: *did2*-*ura3*) in this study was stored at − 80 °C in 30% (v/v) glycerol solution tubes. The construction process is described as follows. After knocked out the ku70, the YL-C11 was constructed by simultaneously replacing the snf gene by *tHMG*-*carB*-*carRA*-*ggs1* and replacing the gut2 gene by *did2*-*ura3* in *Y. lipolytica po1f*. (ATCC # MYA-2613).

The rejuvenation of engineered *Y. lipolytica* stain YL-C11 was carried out on YPD agar medium. A single colony was pre-cultured in the YPD medium at 30 °C, 180 rpm for 24 h. Then 1 mL the pre-cultured solution was transferred to 250 mL shake-flask containing 50 mL medium, which containing (100 mL) 3 g glucose, 1 g casein peptone, 1 g yeast, 0.3 g (NH_4_)_2_SO_4_, 0.25 g KH_2_PO_4_, and 0.05 g MgSO_4_. The seed was incubated at 30 °C, 180 rpm for 48 h.

### Fed-Batch fermentation and DO-stat Fed-batch fermentation

Fed-batch and DO-stat fed-batch fermentations were performed in a 5 L bioreactor (Bioflo 110, New Brunswick, USA) with working volume at 4 L. The medium was identical to the shake flask medium apart from glucose concentration. An aeration rate of 0.25–1.25 vvm, agitation speed was at 400–900 rpm, 30 °C, and pH 5.5 was controlled with 15% ammonia, separately.

In fed-batch fermentation, the glucose was maintained at around 5 g/L. In the early stages of fermentation, when the value of O_2_ was less than 15%, the aeration rate and agitation speed increased alternately until the aeration rate and agitation speed were reached a maximum of 1.25 vvm and 900 rpm.

In DO-stat fed-batch fermentation, the initial glucose concentration was 8 g/L because this concentration allows just made the aeration rate, and agitation speed reached max value. When the aeration rate and agitation speed reached max value, the pure glucose solution was pumped into the fermenter to maintained DO at 10–20%. (NH_4_)_2_SO_4_ (5%) was added at 24^th^, 48^th^, and 72^nd^ hour to avoid pH rise.

## Analytical methods

### Dry cell weight and cell density determination

DCW was calculated as described previously^39^.

### Glucose and β-carotene analysis

The glucose concentration of fermentation broth was measured by an SBA-40C bio-analyzer (Shandong Academy of Sciences; Jinan, China).

The β-carotene was extracted as described previously^40^ and measured by UHPLC (Agilent Technologies 1290 Infinity Series system, USA) using a C18 column (Agilent Poroshell 120 EC-C18 1.9 μm, 2.1 × 50 mm, 699675-902, USJSA03892, B19249) at UV 450 nm. Methanol, acetonitrile, and isopropanol (60:30:10) were used in the mobile phase at a flow rate of 0.8 mL/min at 35 °C.

### Analysis of ATP and NADP/NADPH

The ATP and NADP^+^/NADPH were measured by an ATP assay kit (Beyotime, Nanjing, China) according to the manufacturer’s protocols.

### Transcriptional levels of the related genes in the β-carotene synthesis pathway

Transcriptional levels of the related genes in the β-carotene synthesis pathway were determined by qPCR. The qPCR was carried out using the SYBR tip green qPCR super mix kit (Transgen; Beijing, China). The *rpoZ* gene was used as the internal standard. The *Actin* gene was used as the internal control; the Primer and Sequence used during q-PCR see supplementary Table S1. The relative gene expression analysis was performed using the method published previously^41^.

Accession numbers: opt *CarRA* (KY971027), opt *CarB* (KY971026), *GGS1* (YALI0D17050g), opt *tHMG* (YALI0E04807g).

### Kinetic model

The logistic equation was used to fit the growth kinetics of YL-C11. The Luedeking-Piret equation was used to describe the β-carotene synthesis and glucose consumption. The detailed equations are presented in results of fermentation kinetics of YL-C11.

### Statistics analysis

Data from three replicated trials for each treatment are presented as the format of means with standard deviation. Data in Figs. 3, 4, 5 were analyzed using one-way ANOVA, followed by Duncan’s multiple range test to determine the significant difference between the means using SPSS software. *P* < 0.05 was considered statistically significant. Origin software was used for the statistical analysis and graphs construction.

### Ethical statement

This article does not contain any studies with human participants or animals performed by any of the authors.

## Supplementary information

Supplementary information

## Data Availability

The datasets generated during and/or analyzed during the current study are available from the corresponding author on reasonable request.

## References

[1] Silva HD (2011). Nanoemulsions of β-carotene using a high-energy emulsification–evaporation technique. J. Food Eng..

[2] Patel AS, Kar A, Dash S, Dash SK (2019). Supercritical fluid extraction of β-carotene from ripe bitter melon pericarp. Sci. Rep..

[3] Raja R, Hemaiswarya S, Rengasamy R (2007). Exploitation of Dunaliella for beta-carotene production. Appl. Microbiol. Biotechnol..

[4] Jianming Y, Guo L (2014). Biosynthesis of β-carotene in engineered *E. coli* using the MEP and MVA pathways. Microbial. Cell Fact..

[5] Heinonen OP (1998). Prostate cancer and supplementation with α-tocopherol and β-carotene: incidence and mortality in a controlled trial. JNCI J. Natl. Cancer Inst..

[6] Bogacz-Radomska L, Harasym J (2018). β-Carotene—properties and production methods. Food Qual. Saf..

[7] Gopal K (2015). Attrition of hepatic damage inflicted by angiotensin II with α-tocopherol and β-carotene in experimental apolipoprotein E knock-out mice. Sci. Rep..

[8] Ishida M (2018). Effects of supplemental β-carotene on colostral immunoglobulin and plasma β-carotene and immunoglobulin in Japanese Black cows. Anim. Sci. J..

[9] Nester, R. *Beta-carotene market: global demand analysis & opportunity outlook 2024*. https://www.researchnester.com/reports/beta-carotene-market-global-demand-analysis-opportunity-outlook-2024/267 (2019).

[10] He Z (2017). β-Carotene production promoted by ethylene in *Blakeslea trispora* and the mechanism involved in metabolic responses. Process. Biochem..

[11] Larroude M (2018). A synthetic biology approach to transform *Yarrowia lipolytica* into a competitive biotechnological producer of beta-carotene. Biotechnol. Bioeng..

[12] Wang R (2017). Engineering of β-carotene hydroxylase and ketolase for astaxanthin overproduction in *Saccharomyces cerevisiae*. Front. Chem. Sci. Eng..

[13] Wu T (2017). Membrane engineering: a novel strategy to enhance the production and accumulation of beta-carotene in *Escherichia coli*. Metab. Eng..

[14] Beopoulos A, Desfougéres T, Sabirova J, Nicaud J-M, Timmis KN (2010). Handbook of Hydrocarbon and Lipid Microbiology.

[15] Theron CW, Vandermies M, Telek S, Steels S, Fickers P (2020). Comprehensive comparison of *Yarrowia lipolytica* and *Pichia pastoris* for production of *Candida antarctica* lipase B. Sci. Rep..

[16] Jang IS, Yu BJ, Jang JY, Jegal J, Lee JY (2018). Improving the efficiency of homologous recombination by chemical and biological approaches in *Yarrowia lipolytica*. PLoS ONE.

[17] Kildegaard KR (2017). Engineering of *Yarrowia lipolytica* for production of astaxanthin. Synth. Syst. Biotechnol..

[18] Holland MNACR (1995). Growth kinetics of single-cell protein in batch fermenters. J. Food Eng..

[19] Schultz N, Chang L, Hauck A, Reuss M, Syldatk C (2006). Microbial production of single-cell protein from deproteinized whey concentrates. Appl. Microbiol. Biotechnol..

[20] Li X (2019). High cell density culture of baker's yeast FX-2 based on pH-stat coupling with respiratory quotient. Biotechnol. Appl. Biochem..

[21] Roukas T (1996). Ethanol production from non-sterilized beet molasses by free and immobilized *Saccharomyces cerevisiae* cells using fed-batch culture. J. Food Eng..

[22] Wen S, Zhang T, Tan T (2006). Maximizing production of glutathione by amino acid modulation and high-cell-density fed-batch culture of *Saccharomyces cerevisiae*. Process. Biochem..

[23] Wang Y (2016). Improvement of l-lactic acid productivity from sweet sorghum juice by repeated batch fermentation coupled with membrane separation. Bioresour. Technol..

[24] Hu Z-C, Zheng Y-G, Shen Y-C (2010). Dissolved-oxygen-stat fed-batch fermentation of 1,3-dihydroxyacetone from glycerol by *Gluconobacter oxydans* ZJB09112. Biotechnol. Bioprocess. Eng..

[25] Chia M, Van Nguyen TB, Choi WJ (2008). DO-stat fed-batch production of 2-keto-D-gluconic acid from cassava using immobilized *Pseudomonas aeruginosa*. Appl. Microbiol. Biotechnol..

[26] Bhuvanesh S, Arunkumar C, Kaliraj P, Ramalingam S (2010). Production and single-step purification of *Brugia malayiabundant* larval transcript (ALT-2) using hydrophobic interaction chromatography. J. Ind. Microbiol. Biotechnol..

[27] Woo SH, See-Hyoung P, Hyung-Kwon L, Kyung-Hwan J (2005). Extended operation of a pressurized 75-L bioreactor for shLkn-1 production by *Pichia pastoris* using dissolved oxygen profile control. J. Ind. Microbiol. Biotechnol..

[28] Maghsoudi A (2012). A new methanol-feeding strategy for the improved production of β-galactosidase in high cell-density fed-batch cultures of *Pichia pastoris* Mut+ strains. Biotechnol. Bioprocess. Eng..

[29] Kim BS, Lee SC, Lee SY, Chang YK, Chang HN (2004). High cell density fed-batch cultivation of *Escherichia coli* using exponential feeding combined with pH-stat. Bioprocess. Biosyst. Eng..

[30] Son MK, Hong SJ, Lee YH (2007). Acetate-mediated pH-stat fed-batch cultivation of transconjugant Enterobacter sp. BL-2S over-expressing glmS gene for excretive production of microbial polyglucosamine PGB-1. J. Ind. Microbiol. Biotechnol..

[31] Khatri NK, Hoffmann F (2006). Impact of methanol concentration on secreted protein production in oxygen-limited cultures of recombinant *Pichia pastoris*. Biotechnol. Bioeng..

[32] Van Hoek P, Van Dijken JP, Pronk JT (1998). Effect of specific growth rate on fermentative capacity of baker’s yeast. Appl. Environ. Microbiol..

[33] Picotto LD (2017). An effective and simplified DO-stat control strategy for production of rabies glycoprotein in *Pichia pastoris*. Prot. Expr. Purif..

[34] Kawaguchi H (2019). Enhanced phenyllactic acid production in *Escherichia coli *via oxygen limitation and shikimate pathway gene expression. Biotechnol. J..

[35] Cruz MV, Gouveia AR, Dionísio M, Freitas F, Reis MAM (2019). A process engineering approach to improve production of P(3HB) by *Cupriavidus necator* from used cooking oil. Int. J. Polym. Sci..

[36] Song P (2013). Two-stage oxygen supply strategy for enhanced lipase production by *Bacillus subtilis *based on metabolic flux analysis. Biochem. Eng. J..

[37] Martinez I, Bennett GN, San KY (2010). Metabolic impact of the level of aeration during cell growth on anaerobic succinate production by an engineered *Escherichia coli* strain. Metab. Eng..

[38] Xu H (2009). A two-stage oxygen supply strategy for enhanced l-arginine production by *Corynebacterium crenatum* based on metabolic fluxes analysis. Biochem. Eng. J..

[39] Wen S, Zhang T, Tan T (2005). Optimization of the amino acid composition in glutathione fermentation. Process. Biochem..

[40] Gao S (2017). Iterative integration of multiple-copy pathway genes in *Yarrowia lipolytica* for heterologous β-carotene production. Metab. Eng..

[41] Su A (2018). Metabolic redesign of *Rhodobacter sphaeroides* for lycopene production. J. Agric. Food Chem..

