# Supplementary material for: Dissolved-oxygen feedback control fermentation for enhancing β-carotene in engineered Yarrowia lipolytica
Source: Sci Rep. 2020 Oct 13;10:17114. doi: 10.1038/s41598-020-74074-0 (PMC7555900; doi:10.1038/s41598-020-74074-0)
Supplement: Supplementary file 1 — Supplementary information [file 41598_2020_74074_MOESM1_ESM.docx]

**Dissolved-oxygen feedback control fermentation for enhancing β-carotene in engineered *Yarrowia lipolytica***

Peng Jun Lv ^a^, Shan Qiang^b^, Liang Liu^a^, Ching Yuan Hu^a^, Yong Hong Meng^a,*^

a, Engineering Research Center of High Value Utilization of Western China Fruit Resources, Ministry of Education; National Research & Development Center of Apple Processing Technology; College of food engineering and nutritional science, Shaanxi Normal University, 620 West Changan Avenue, Changan, Xian 710119, P.R. China

b, Xian Healthful Biotechnology Co., Ltd. Hang Tuo Road, Changan, Xian 710100, P. R. China

**Supplementary Information**


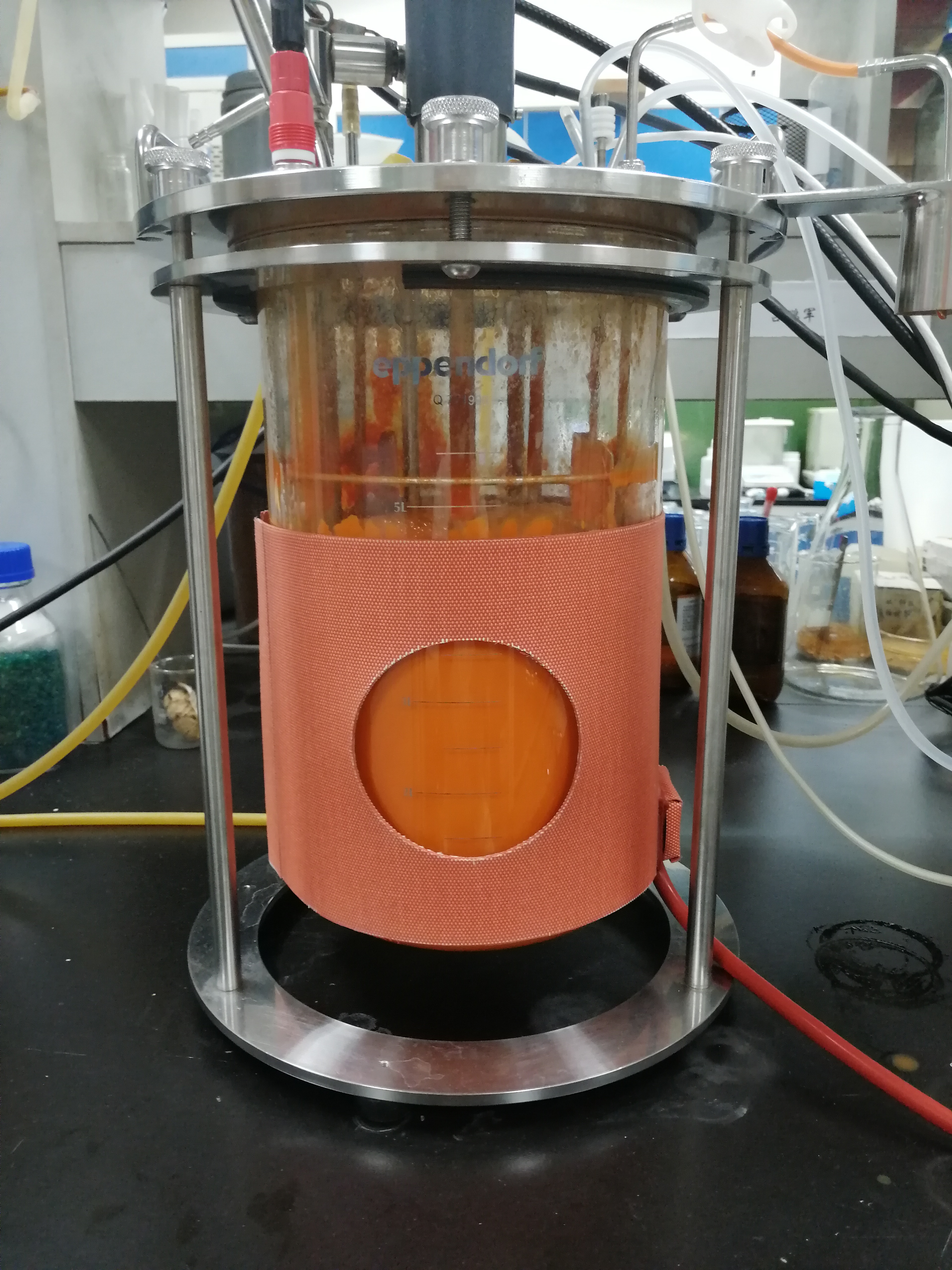


Supplementary Fig S1. Picture of fermentation


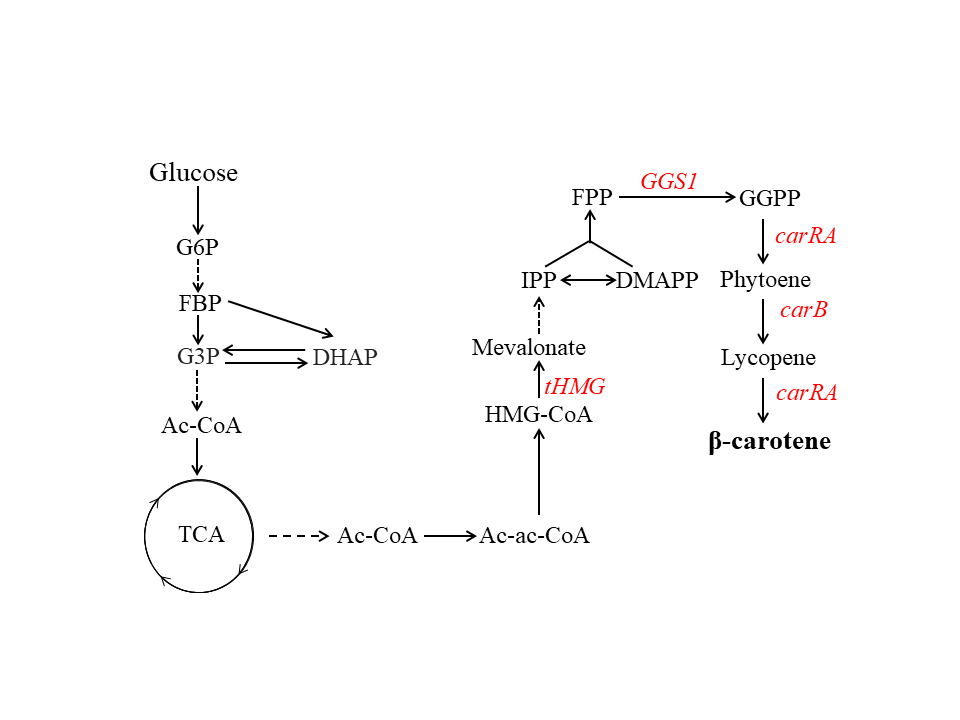


Supplementary Fig S2. Scheme of metabolic pathways leading to the production of β-carotene in YL-C11. (tHMG, GGS1,carRA and carB are four crucial genes in the β-carotenoid synthesis pathway)

Supplementary Fig S3. UHPLC of β-carotene (standard substances)

Supplementary Fig S4. UHPLC of β-carotene (sample)

Supplementary Tab. S1. The Primer and Sequence used during q-PCR

| Primer | Sequence |
| --- | --- |
| tHmg-F | CTCAGGACGGTATGACACGA |
| tHmg-R | GAGTTGAAGGCCTTTCGCAT |
| GGS1-F | ATCAAGGTGGACAAGAGVGA |
| GGS1-R | ATCAAGGTGGACAAGAGCGA |
| carRA-F | GCTCTGCTGGCTATCACCTA |
| carRA-R | GTCGTCGCAGAATGTACTGC |
| carB-F | GACAAGGACAAGCGAGTGAC |
| carB-R | CAGGGTCTTCTTGGTCCAGT |
